# A Text-Book of Practical Therapeutics

**Published:** 1899-03

**Authors:** 


**A Text-Book of Practical Therapeutics.** By Hobart Amory Hare, M.D. Sixth Edition. Pp. 758. London: Henry Kimpton. 1897.

Seated in his editorial chair, Dr. H. A. Hare must see pass before him in review the vast crowd of therapeutical experiences and suggestions which is ever being recorded in the medical journals. That he has long since brought out a successful text-book of therapeutics is therefore not to be wondered at; this work in its present edition has been largely re-written, to bring it up more fully abreast with the present state of the art of which it treats. The two more important sections of the book are: (1) A description of the properties and uses of drugs, arranged in alphabetical order—a plan now adopted in most text-books of Materia Medica; and (2) A list of the principal recognised diseases, also arranged alphabetically, giving a succinct yet sometimes fairly full account of the accepted treatment for each disease. Although Dr. Hare is a Professor of Therapeutics, as well as editor of a journal devoted to the same subject, he is not unduly carried away by every ephemeral novelty. In the preface he says: "Only those measures which have proved useful and reliable in therapeutics are included." An examination of the text of the book certainly corroborates this statement, and we should indeed have been not sorry if some additional suggestions had here and there added to the well-tried measures enumerated. The article on the treatment of phthisis is almost depressing in its negations.

The book is excellently printed, wonderfully free from clerical or typographical errors, and is elaborately indexed. Everything that is affirmed in the book can be accepted as trustworthy, and altogether the work is a bright and worthy monument of the author's industry and learning.

